# Burden of Hospital Acquired Infections and Antimicrobial Use in Vietnamese Adult Intensive Care Units

**DOI:** 10.1371/journal.pone.0147544

**Published:** 2016-01-29

**Authors:** Vu Dinh Phu, Heiman F. L. Wertheim, Mattias Larsson, Behzad Nadjm, Quynh-Dao Dinh, Lennart E. Nilsson, Ulf Rydell, Tuyet Thi Diem Le, Son Hong Trinh, Hung Minh Pham, Cang Thanh Tran, Hanh Thi Hong Doan, Nguyen Thua Tran, Nhan Duc Le, Nhuan Van Huynh, Thao Phuong Tran, Bao Duc Tran, Son Truong Nguyen, Thao Thi Ngoc Pham, Tam Quang Dang, Chau Van Vinh Nguyen, Yen Minh Lam, Guy Thwaites, Kinh Van Nguyen, Hakan Hanberger

**Affiliations:** 1 Intensive Care Unit, National Hospital for Tropical Diseases, Ha Noi, Vietnam; 2 Wellcome Trust Major Overseas Programme, Oxford University Clinical Research Unit, Hanoi, Vietnam; 3 Centre for Tropical Medicine, Nuffield Department of Clinical Medicine, University of Oxford, United Kingdom; 4 Global Health (IHCAR), Karolinska Institutet, Stockholm, Sweden; 5 Clinical Microbiology, Department of Clinical and Experimental Medicine, Linköping University, Linköping, Sweden; 6 Infectious Diseases, Department of Clinical and Experimental Medicine, Linköping University, Linköping, Sweden; 7 Intensive Care Unit, Bach Mai Hospital, Ha Noi, Vietnam; 8 Board of Directors, Viet Duc Hospital, Ha Noi, Vietnam; 9 Pharmacy, Saint-Paul Hospital, Ha Noi, Vietnam; 10 Intensive Care Unit, Viet Tiep Hospital, Hai Phong, Vietnam; 11 Board of Directors, Vietnam-Sweden Uong Bi Hospital, Quang Ninh, Vietnam; 12 Department of General Internal Medicine & Geriatric, Hue Central General Hospital, Hue, Vietnam; 13 Board of Directors, Da Nang Hospital, Da Nang, Vietnam; 14 Infectious Department, Binh Dinh Hospital, Binh Dinh, Vietnam; 15 Intensive Care Unit, Khanh Hoa Hospital, Khanh Hoa, Vietnam; 16 Planning Department, Dak Lak Hospital, Dak Lak, Vietnam; 17 Board of Directors, Cho Ray Hospital, Ho Chi Minh City, Vietnam; 18 Board of Directors, Can Tho Central General Hosptial, Can Tho, Vietnam; 19 Board of directors, Hospital for Tropical Diseases, Ho Chi Minh City, Vietnam; 20 Wellcome Trust Major Overseas Programme, Oxford University Clinical Research Unit, Ho Chi Minh City, Vietnam; 21 Board of Directors, National Hospital for Tropical Diseases, Ha Noi, Vietnam; University Hospital San Giovanni Battista di Torino, ITALY

## Abstract

**Background:**

Vietnam is a lower middle-income country with no national surveillance system for hospital-acquired infections (HAIs). We assessed the prevalence of hospital-acquired infections and antimicrobial use in adult intensive care units (ICUs) across Vietnam.

**Methods:**

Monthly repeated point prevalence surveys were systematically conducted to assess HAI prevalence and antimicrobial use in 15 adult ICUs across Vietnam. Adults admitted to participating ICUs before 08:00 a.m. on the survey day were included.

**Results:**

Among 3287 patients enrolled, the HAI prevalence was 29.5% (965/3266 patients, 21 missing). Pneumonia accounted for 79.4% (804/1012) of HAIs Most HAIs (84.5% [855/1012]) were acquired in the survey hospital with 42.5% (363/855) acquired prior to ICU admission and 57.5% (492/855) developed during ICU admission. In multivariate analysis, the strongest risk factors for HAI acquired in ICU were: intubation (OR 2.76), urinary catheter (OR 2.12), no involvement of a family member in patient care (OR 1.94), and surgery after admission (OR 1.66). 726 bacterial isolates were cultured from 622/1012 HAIs, most frequently *Acinetobacter baumannii* (177/726 [24.4%]), *Pseudomonas aeruginosa* (100/726 [13.8%]), and *Klebsiella pneumoniae* (84/726 [11.6%]), with carbapenem resistance rates of 89.2%, 55.7%, and 14.9% respectively. Antimicrobials were prescribed for 84.8% (2787/3287) patients, with 73.7% of patients receiving two or more. The most common antimicrobial groups were third generation cephalosporins, fluoroquinolones, and carbapenems (20.1%, 19.4%, and 14.1% of total antimicrobials, respectively).

**Conclusion:**

A high prevalence of HAIs was observed, mainly caused by Gram-negative bacteria with high carbapenem resistance rates. This in combination with a high rate of antimicrobial use illustrates the urgent need to improve rational antimicrobial use and infection control efforts.

## Introduction

Hospital-acquired infections (HAIs) and antimicrobial resistance are growing global public health problems [1,2]. The incidence of HAIs is substantially higher in Low and Middle Income Countries (LMICs), with an average prevalence of 15.5%, compared to prevalence of 7.1% and 4.5% in Europe and USA, respectively [3]. This problem is more serious in intensive care units (ICUs). The HAI prevalence in ICUs ranges from 9.1% in the United States to about 23.0%-23.5% in Europe and England [4–7], and even higher in LMICs with a pooled prevalence of 35.2% [1]. A recent report of the International Nosocomial Infection Control Consortium 2007–2012 from 503 ICUs shows that ventilator-associated pneumonia is fifteen times and catheter-associated urinary tract infection four times higher in LMICs than in better resourced settings [8]. Due to economic development in LMICs, the healthcare systems are changing rapidly, with increasing ICU capacities. However resource constraints often result in high occupancy rates, crowding, a lack of isolation facilities, and insufficient resources for adequate infection control all of which may contribute to the reported high incidence of HAIs and drug-resistant infections at ICU’s in these settings [1,9,10].

Vietnam is a LMIC with a population of 90.796 million [11] and an increasingly sophisticated health care system, typical of countries in the region. Health expenditure per capita in Vietnam was around 100$ per annum in 2012, approximately a seventh of the regional average [11]. Up to now, there is no national surveillance system for HAIs and limited data about HAIs in ICUs. The few studies performed are small and only some include ICUs, but reported that the HAI prevalence in those ICUs ranged from 19.3% to 31.3% [12–17]. Only one of these studies is from the international peer reviewed literature [12], the others are published in the Vietnamese medical literature. Antimicrobial resistance levels are high in Vietnam; up to 70% of *Enterobacteriaceae* were resistant to 3^rd^ generation cephalosporins and > 40.0% of *Acinetobacter* spp. resistant to carbapenems in 2009 [9,18].

In order to provide up-to-date, systematic data and to demonstrate the feasibility of developing a national surveillance network for ICUs in a LMIC, we performed a prospective study on the prevalence of HAI in ICUs across Vietnam, exploring risk factors, antimicrobial use, and antimicrobial resistance [19].

## Materials and Methods

### Study design, hospital and patient selection

**Fig 1:**
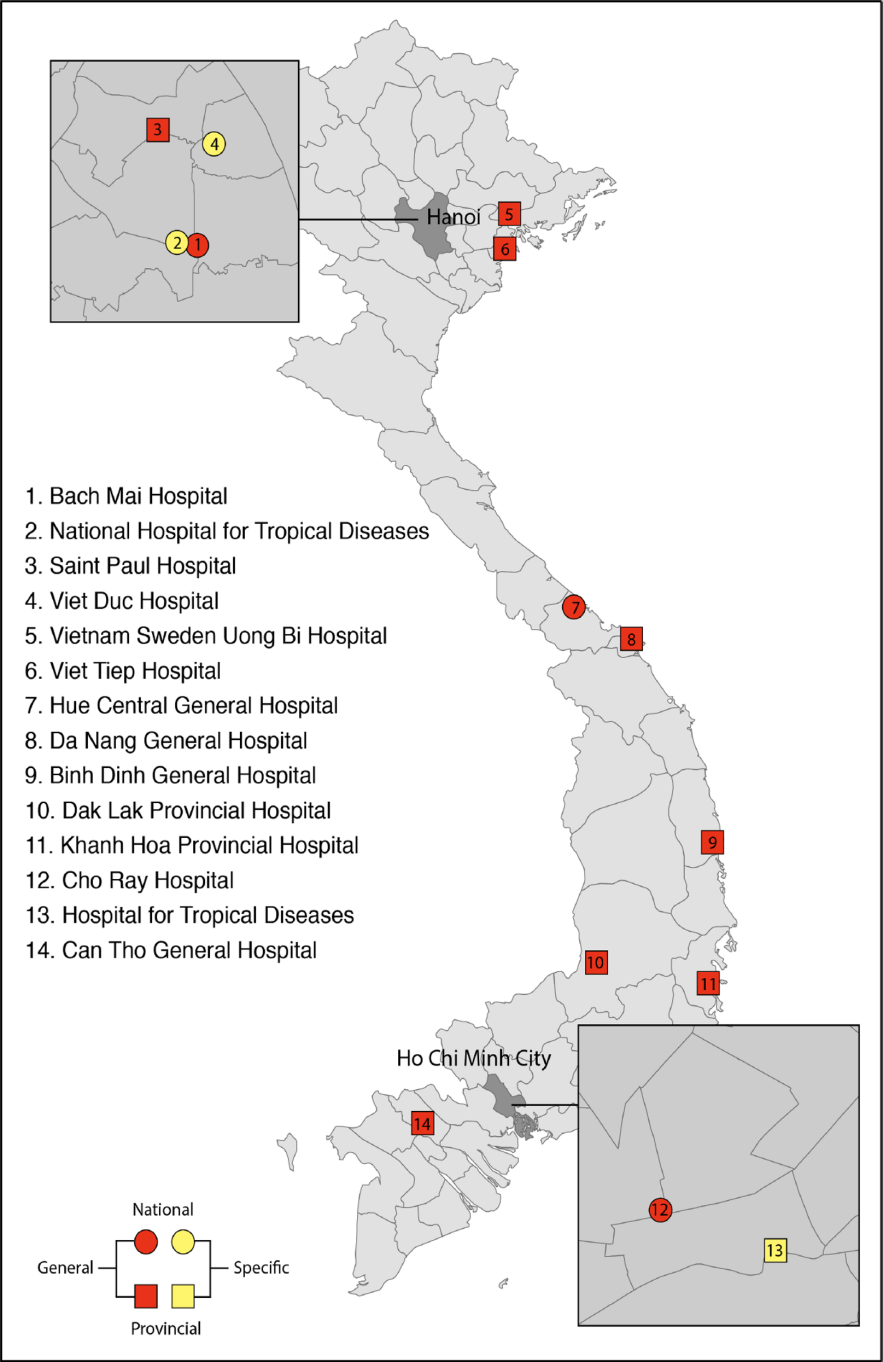
The Study Site Locations.

We conducted a repeated point prevalence survey (PPS) to determine the prevalence of HAIs, and to assess antimicrobial use and antimicrobial resistance using the methodology developed by the European Center for Disease Prevention and Control (ECDC) [20]. The survey was conducted on one day each month from October 2012 through September 2013 in 15 adult ICUs in 14 acute care hospitals, of which 7 were tertiary hospitals and 7 provincial hospitals, throughout Vietnam (Fig 1). Patients aged ≥ 18 years, admitted to participating ICUs before 8 a.m. on the survey day, and remaining there at the survey time were included regardless after that time patient was discharged or remain in that ICU.

### Data collection

The following patient data were collected: reason for admission, location of patient at admission to ICU, comorbidity, current interventions, involvement of patient’s family in patient care (participating in bathing, cleaning, changing position, and feeding patients), antimicrobial agent use for any indication, presence of HAI according to ECDC definitions [20], and results of routine microbiological investigations.

All participating hospitals provided data on basic infrastructure and infection control indicators at the beginning of the study, including total number of beds, rooms, single bed rooms, number of doctors and nurses at the ICUs, admissions per year, patient days per year, alcohol hand rub consumption, and availability of alcohol hand rub at ICU bed.

All participating hospital laboratories were trained to follow the Clinical and Laboratory Standards Institute guidelines (CLSI) for antimicrobial susceptibility testing and were enrolled in an external quality assurance program (The United Kingdom National External Quality Assessment Service (UK NEQAS) for Microbiology) [19]. All participating doctors received training on the protocol, HAI definitions, how to complete case record forms, and data entry before the study. A database adapted from *HELICSWin*.*Net* developed by the ECDC was used for the study [21]. Each ICU received a laptop with the software installed and Vietnamese instructions. The anonymised data were submitted to the project coordinators electronically once a month during the implementation period. Data were then checked for missing or inconsistent data, with regular queries sent to the hospitals and visits by study investigators to assist in data reconciliation.

### Statistical analysis

For descriptive statistics we calculated percentage, frequency, mean, and median values and 95% confidence interval (95% CI) or interquartile ranges (IQR), as appropriate. Where patients were included in more than one PPS, we only used the data from the first survey and had no HAI at ICU admission to calculate the odds ratios of risk factors for developing HAIs in ICU. After univariate analysis, we included all HAI risk factors in the multivariate logistic regression models. We used IBM SPSS Statistics software (version 22 IBM, California, USA) for data analysis. P-values < 0.05 (two-sided) were considered statistically significant.

### Ethical considerations

The Ethical Committee of the National Hospital for Tropical Diseases (27/HDDD-NHTD) had approved of the study and confirmed that the need for informed consent was waived due to data were anonymous and collected by surveillance and no intervention was conducted; and the study was also approved by the Vietnamese Ministry of Health (4921/QD-BYT).

## Results

### Hospital and ICU resources

Hospital sizes ranged from 280 to 2362 beds (median 950; IQR 750–1650) and the participating ICU sizes ranged from 10 to 60 beds (median 20; IQR 18–31). Mean length of ICU stays per individual ICU ranged from 3.8 to 16.0 days with a median mean length of stay across the ICUs of 6.4 days (IQR: 4.8–9.3). There were no national antimicrobial therapy guidelines, but locally derived guidelines were used at 7 of 15 ICUs. In each ICU one nurse took care of 1.9 to 5.9 beds (median 3.6, IQR 3.1–4.4 beds) per 8 hours shift. During working hours, there was a median of 3.5 doctors (IQR: 2.5–4.8) for 10 ICU beds. Alcohol hand rub was available at the bedside in all participating ICUs. Median alcohol hand rub consumption per patient day was 66.4 ml (IQR: 23.7–100.5). More details are presented in Table A in S1 File.

### Patient characteristics

In total, 3,401 patients were screened, of whom 3287 (114 were excluded due to age < 18 years) were enrolled from 15 ICUs of 14 participating hospitals between October 2012 and October 2013. Due to prolonged ICU stay, 162 patients were enrolled in more than one survey leaving 3125 unique patients.

The median age was 61.0 years (IQR 45.0–77.0) and 63.9% (2101/3287) of patients were male. Most patients (46.2%; 1427/3088; 199 missing) were admitted directly from the community, while 30.1% (930/3088) of the patients entered the ICU from another department in the same hospital and 20.7% (638/3088) were referred from other hospitals. 52.3% (1719/3287) patients were intubated, 28.0% (921/3287) had a central vascular catheter, 49.2% (1616/3287) had a urinary catheter, and 8.2% (270/3287) were on renal replacement therapy. In 63.0% (2072/3287) of patients, family members were involved in patient care. Details are presented in Table B in S1 File.

Comorbidity was present in 39.6% of patients (1249/3151, 136 missing), of which 190 patients had two or more comorbidities. Common comorbidities were: stroke sequelae 27.5% (343/1249), diabetes mellitus 24.8% (310/1249), chronic obstructive pulmonary disease 20.7% (259/1249), renal failure 11.5% (144/1249), harmful alcohol use 11.3% (141/1249), active malignancy 9.5% (119/1249), and induced immunosuppression 6.1% (76/1249).

### HAI prevalence

Overall, 29.5% (965/3266 patients, 21 missing) had at least one HAI; 922 patients had one HAI, 39 patients had 2 HAIs, and 4 patients had 3 HAIs. The HAI prevalence ranged widely between ICUs from 5.6% to 60.9% with a median prevalence of 30.5% (Table B in S1 File). Prevalence of HAI per month in year fluctuated from 23.8% in April to 34.8% in November, prominent in March (34.1%), July (33.4%), November (34.8%), and December (34.7%) (Table C in S1 File). Pneumonia was the most common HAI (79.4% [804/1012]), followed by blood stream infection (4.4% [44/1012]), and surgical site infections (4.2% [42/1012]). Most HAIs (84.5% [855/1012]) were acquired in the survey hospital: 42.5% (363/855) acquired prior to ICU admission and 57.5% (492/855) developed after ICU admission. The median time from hospital admission to diagnosis of HAI was 7 days (IQR: 3–15 days). Device-associated HAIs accounted for 643/1012 (63.5%) of HAIs, mainly pneumonia (589/643 [91.6% of device-associated HAIs]) (Table 1).

**Table 1:** Location of Acquired HAI and Association with Invasive Devices.

Type of infections	Location Acquired HAI				Related device			Total No. of HAI, n (%)
	Current hospital		Other hospital	UNK	Yes	No	UNK	
	In ICU	Out ICU						
**Pneumonia & LRTI**^**a**^**, n (%)**	389 (48.4)	301 (37.4)	60 (7.5)	54 (6.7)	589 (73.3)	120 (14.9)	95 (11.8)	804 (79.4)
**Bloodstream infection, n (%)**	34 (77.3)	8 (18.2)	2 (4.5)	0	27 (61.4)	11 (25.0)	6 (13.6)	44 (4.4)
**Surgical site infection, n (%)**	23 (54.8)	11 (26.2)	6 (14.3)	2 (4.8)	NA	NA	NA	42 (4.2)
**Gastrointestinal infection, n (%)**	13 (32.5)	19 (47.5)	7 (17.5)	1 (2.5)	NA	NA	NA	40 (4.0)
**Urinary tract infection, n (%)**	18 (62.1)	8 (27.6)	3 (10.3)	0	27 (93.1)	2 (6.9)	0	29 (2.9)
**Central nervous system infection, n (%)**	5 (26.3)	4 (21.1)	8 (42.1)	2 (10.5)	NA	NA	NA	19 (1.9)
**Skin and soft tissue infection, n (%)**	2 (13.3)	6 (40.0)	1 (6.7)	6 (40.0)	NA	NA	NA	15 (1.5)
**Other infections**^**b**^**, n (%)**	8 (42.1)	6 (31.6)	2 (10.5)	3 (15.8)	NA	NA	NA	19 (1.9)
**Total, n (%)**	492 (48.6)	363 (35.9)	89 (8.8)	68 (6.7)	643 (63.5)	133 (13.1)	101 (10.0)	1012 (100)

UNK: unknown or missing data

NA: Not applicable

LRTI: low respiratory tract infection

^a^ Only one case of LRTI

^b^ Including: nine cases of systemic infection, four catheter related infections, four reproductive tract infections, one endocarditis and one eyes ear nose throats infection not classified.

Note: Twenty-one patients whose infection could not be differentiated between community-acquired and hospital-acquired infection were excluded from calculating HAI prevalence.

### Risk factors for HAIs Acquired in ICU

We assessed risk factors for ICU-acquired HAIs in 2,618 patients who were enrolled in the study for the first time and had no HAI at ICU admission. The prevalence of HAI among these patients at survey time was 16.2% (424/2618 patients). In the univariate analysis, exception for gender, age group, and comorbidity, all other factors had risk for HAI with statistically significant. The highest risk factors were intubation (OR 6.31 [95% CI 4.86–8.18]), having surgery after admission (minor surgery OR 4.78 [95% CI 3.57–6.40], major surgery OR 3.78 [95% CI 2.90–4.93]), and urinary catheter (OR 3.90 [95% CI 3.09–4.92]) (Table 2).

**Table 2:** Risk Factors for Acquiring HAI in ICU.

Risk factors	No. of patients (total = 2618), n	Patients with HAI (total = 424), n (%)	Univariate analysis		Multivariate analysis	
			OR (95% CI)	P value	OR (95% CI)	P value
**Gender**						
Male	1656	271 (16.4)	*1*.*03 (0*.*83–1*.*28)*	0.758	1.22 (0.94–1.59)	0.137
Female	962	153 (15.9)	*Reference*		*Reference*	
Missing	0				141	
**Age group**						
18–60 years	1344	202 (15.0)	*Reference*		*Reference*	
> 60 years	1274	222 (17.4)	*1*.*19 (0*.*97–1*.*47)*	0.097	1.17 (0.90–1.52)	0.233
Missing	0				141	
**Location of patients at admission to ICU**						
Community and others	1352	144 (10.7)	*Reference*		*Reference*	
Same hospital	655	171 (26.1)	*2*.*96 (2*.*32–3*.*79)*	< 0.001	1.49 (1.09–2.04)	0.013
Other hospital	507	109 (21.5)	*2*.*30 (1*.*75–3*.*02)*	< 0.001	1.23 (0.87–1.72)	0.239
Missing	104				141	
**Reason for admission**						
Medical disease	1029	120 (11.7)	*Reference*		*Reference*	
Infectious disease	1106	214 (19.3)	*1*.*82 (1*.*43–2*.*31)*	< 0.001	0.91 (0.67–1.23)	0.536
Surgery	368	90 (24.5)	*2*.*45 (1*.*81–3*.*32)*	< 0.001	0.73 (0.45–1.19)	0.214
Missing	115				141	
**Comorbidity**						
No comorbidity	1560	250 (16.0)	*Reference*		*Reference*	
Have comorbidity	972	171 (17.6)	*1*.*12 (0*.*90–1*.*38)*	0.303	0.90 (0.70–1.17)	0.445
Missing	86				141	
**Surgery after admission**						
Non surgery	2009	219 (10.9)	*Reference*		*Reference*	
Minor surgery	252	93 (36.9)	*4*.*78 (3*.*57–6*.*40)*	< 0.001	1.67 (1.16–2.41)	0.005
Major surgery	354	112 (31.6)	*3*.*78 (2*.*90–4*.*93)*	< 0.001	1.66 (1.06–2.58)	0.025
Missing	3				141	
**Intubation**						
Yes	1252	346 (27.6)	*6*.*31 (4*.*86–8*.*18)*	< 0.001	2.76 (2.03–3.75)	< 0.001
No	1366	78 (5.7)	*Reference*		*Reference*	
Missing	0				141	
**Central vascular catheter**						
Yes	693	197 (28.4)	*2*.*97 (2*.*39–3*.*68)*	< 0.001	1.47 (1.05–2.06)	0.026
No	1925	227 (11.8)	*Reference*		*Reference*	
Missing	0				141	
**Urinary catheter**						
Yes	1226	312 (25.4)	*3*.*90 (3*.*09–4*.*92)*	< 0.001	2.12 (1.57–2.87)	< 0.001
No	1392	112 (8.0)	*Reference*		*Reference*	
Missing	0				141	
**Hemodialysis**						
Yes	203	51 (25.1)	*1*.*84 (1*.*31–2*.*57)*	< 0.001	1.34 (0.87–2.08)	0.188
No	2415	373 (15.4)	*Reference*		*Reference*	
Missing	0				141	
**Peripheral vascular catheter**						
Yes	2202	321 (14.6)	*0*.*52 (0*.*40–0*.*67)*	< 0.001	1.47 (1.02–2.12)	0.037
No	416	103 (24.8)	*Reference*		*Reference*	
Missing	0				141	
**Family involved patient care**						
Yes	1717	190 (11.1)	*Reference*		*Reference*	
No	901	234 (26.0)	*2*.*82 (2*.*28–3*.*48)*	< 0.001	1.94 (1.49–2.51)	< 0.001
Missing	0				141	
**Each day longer from ICU admission to survey time**						
In analysis	2513	424 (16.9)	1.08 (1.07–1.10)	< 0.001	1.08 (1.07–1.10)	< 0.001
Missing	105				141	

For multivariate regression analysis:
Omnibus test of model coefficients: -2 Log likelihood 1720.120, Chi-square (538.055, df 16, p < 0.001) Nagelkerke R-Square: 0.326 Hosmer & Lemeshow test (Chi-square 10.592, df 8, p = 0.226) Classification accuracy: 85.3% (97.0% for no HAI, 28.0% for HAI, with cut value is 0.50).

Multivariate logistic regression identified eight risk factors independently associated with HAIs, which included: intubation (OR 2.76 [95% CI 2.03–3.75]), urinary catheter (OR 2.12 [95% CI 1.57–2.87]), no involvement of a family member in patient care (OR 1.94 [95% CI 1.49–2.51]), surgery after admission (minor surgery OR 1.67 [95% CI 1.16–2.41], major surgery OR 1.66 [95% CI 1.06–2.58]), admission to ICU from the same hospital OR 1.49 [95% CI 1.09–2.04]), central vascular catheter (OR 1.47 [95% CI 1.05–2.06]), peripheral vascular catheter showed a protective effect in univariate analysis but was associated with a significant increased risk for HAIs in multivariate analysis (OR 1.47 [95% CI 1.02–2.12]), and every one day longer of ICU stay (OR 1.08 [95% CI 1.07–1.10]) (Table 2).

### Microbiological Aetiology of HAIs

From 593/965 (61.5%) patients with HAI, 726 microorganisms were reported in association with 622 HAIs. For 390 HAIs in 372 patients no pathogens were isolated. All reported pathogens are summarized in Table 3. Antimicrobial resistance to frequently used antimicrobials was common. Carbapenem resistance was most common in *Acinetobacter baumannii* (89.2% [149/167]) and *Pseudomonas aeruginosa* (55.7% [49/88]). Slightly over 5% of the Enterobacteriaceae isolated were carbapenem resistant, while 14.9% (11/74) *Klebsiella pneumonia* were carbapenem resistant. More than 75% of the *Staphylococcus aureus* isolates were methicillin resistant and more than 57% of the Enterococci were resistant to glycopeptides.

**Table 3:** Microorganisms Causing HAIs.

Name of Microorganisms	All pathogen isolated (total = 726) n (%)	Pathogen for pneumonia (total = 587) n (%)	Pathogen for blood stream infections (total = 44) n (%)	Pathogen for surgical site infections (total = 34) n (%)	Pathogen for urinary tract infections (total = 25) n (%)
**Gram-negative bacteria**	**611 (84.2)**	**516 (87.9)**	**28 (63.6)**	**25 (73.5)**	**15 (60.0)**
*Acinetobacter baumannii*	177 (24.4)	151 (25.7)	10 (22.7)	2 (5.9)	5 (20.0)
*Pseudomonas aeruginosa*	100 (13.8)	92 (15.7)	2 (4.5)	3 (8.8)	0
*Klebsiella pneumoniae*	84 (11.6)	68 (11.6)	5 (11.4)	8 (23.5)	1 (4.0)
*Acinetobacter* spp.	46 (6.3)	46 (7.8)	0	0	0
*Escherichia coli*	39 (5.4)	20 (3.4)	6 (13.6)	4 (11.8)	3 (12.0)
*Enterobacteriaceae*	36 (5.0)	26 (4.4)	1 (2.3)	2 (5.9)	5 (20.0)
*Klebsiella* spp.	35 (4.8)	26 (4.4)	2 (4.5)	2 (5.9)	1 (4.0)
*Providencia* spp.	28 (3.9)	25 (4.3)	0	2 (5.9)	0
*Klebsiella oxytoca*	19 (2.6)	18 (3.1)	0	1 (2.9)	0
*Achromobacter* spp.	18 (2.5)	17 (2.9)	0	1 (2.9)	0
*Stenotrophomonas maltophilia*	6 (0.8)	6 (1.0)	0	0	0
Gram-negative bacilli others	18 (2.5)	17 (2.9)	1 (2.3)	0	0
Gram-negative cocci other	4 (0.6)	4 (0.7)	0	0	0
Anaerobic bacilli	1 (0.1)	0	1 (2.3)	0	0
**Gram-positive bacteria**	**104 (14.3)**	**65 (11.1)**	**15 (34.1)**	**9 (26.5)**	**7 (28.0)**
*Staphylococcus aureus*	39 (5.4)	28 (4.8)	6 (13.6)	3 (8.8)	0
*Staphylococcus* spp.	19 (2.6)	11 (1.9)	6 (13.6)	0	1 (4.0)
*Streptococcus* spp.	11 (1.5)	9 (1.5)	0	1 (2.9)	0
*Enterococcus* spp.	16 (2.2)	9 (1.5)	2 (4.5)	1 (2.9)	2 (8.0)
*Enterococcus faecalis*	8 (1.1)	2 (0.3)	1 (2.3)	3 (8.8)	2 (8.0)
*Enterococcus faecium*	5 (0.7)	0	0	1 (2.9)	2 (8.0)
Gram-positive bacilli other	6 (0.8)	6 (1.0)	0	0	0
**Fungi**	**11 (1.5)**	**6 (1.0)**	**1 (2.3)**	**0**	**3 (12.0)**
*Candida* spp.	10 (1.4)	5 (0.8)	1 (2.3)	0	3 (12.0)
Other fungi	1 (0.1)	1 (0.2)	0	0	0

### Antimicrobial use

Antimicrobials use was evaluated in all enrolled patients. The proportion of patients receiving antimicrobials at survey time ranged from 50.0% to 99.8% per ICU, with a pooled proportion of 84.8% (2787/3287). 733 (22.3%) patients were prescribed one, 1343 (40.8%) two, 552 (16.8%) three, and 159 (4.8%) four antimicrobial agents. The main indications for antimicrobial use were community-acquired infections 41.8% (2386/5711) and HAIs 33.9% (1937/5711). As infectious diseases are common in Vietnam, having a severe community-acquired infection was a common reason for admission to an ICU. Prophylaxis and other use were the indications for antimicrobial use in 9.4% (536/5711) and the indication was unknown in 14.9% (852/5711).

**Fig 2:**
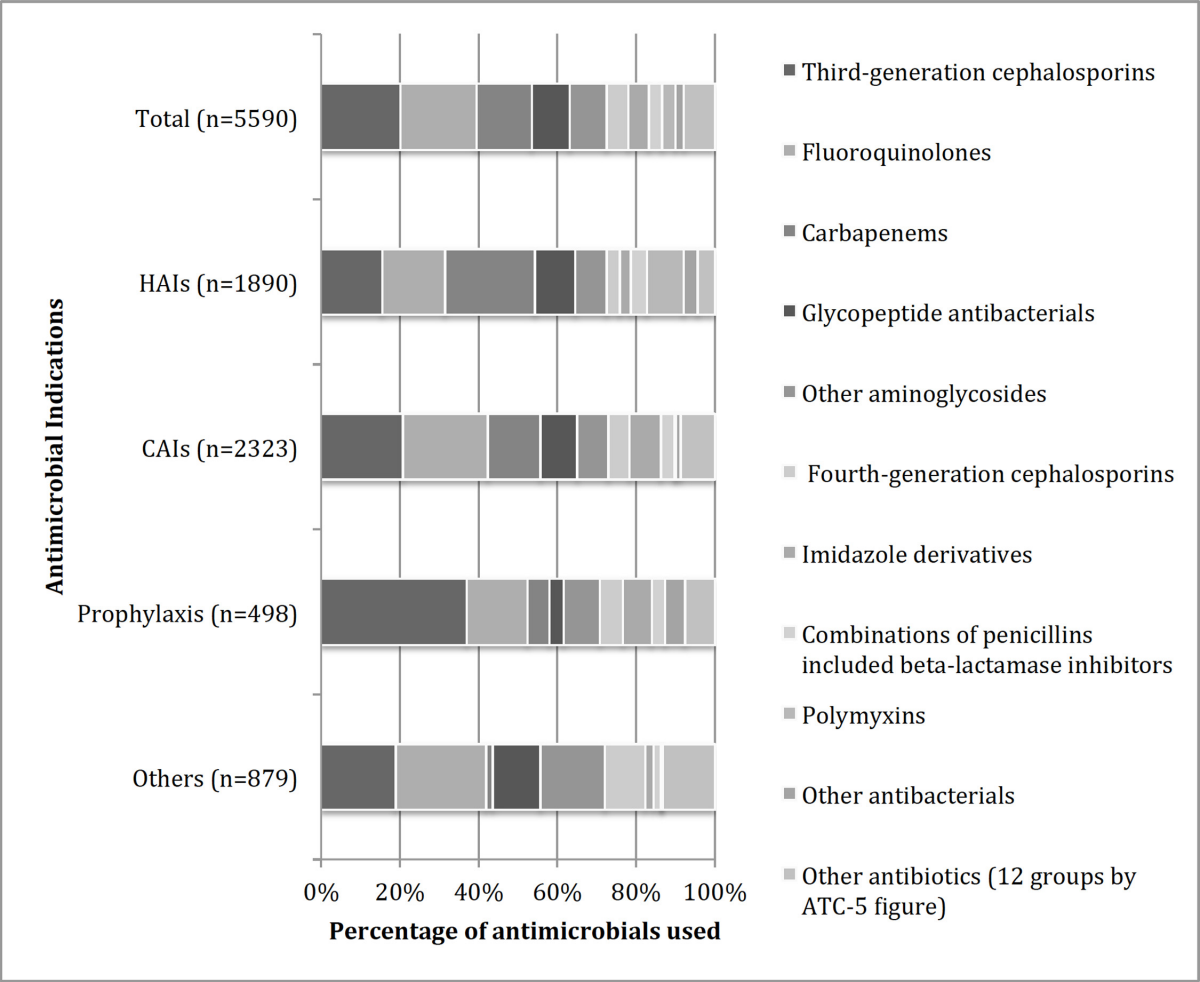
Antimicrobials for Systemic Use by Indication. CAIs: Community acquired infections; HAIs: Hospital-acquired infections; Prophylaxis: Medical and surgical prophylaxis; others: other indications (e.g. erythromycin use for prokinetic) and unknown indication.

Antimicrobials for systemic use (ATC group J01) accounted for 97.9% (5590/5711) of the total antimicrobials used. Of which, third generation cephalosporins, fluoroquinolones, and carbapenems were used most common—accounting for 20.1% (1126/5590), 19.4% (1082/5590), and 14.1% (786/5590), respectively. For the treatment of HAIs, the most frequently used agents were carbapenems at 22.9% (432/1890), fluoroquinolones 16.0% (302/1890), and 3rd generation cephalosporins 15.5% (293/1890). Polymyxins (parenteral colistin) accounted for 3.3% (186/5590) of general use but were the fifth most frequently used agent for HAIs; (Fig 2). For HAIs associated with carbapenem resistant *A*. *baumannii* and *P*. *aeruginosa*, colistin was given to 65.1% (97/149) and 30.6% (15/49), respectively. More details in Tables D and E in S1 File.

## Discussion

The HAI prevalence in this study (29.5%, 965/3266 patients) is higher than that reported from adult ICUs in European hospitals using the same protocol (23.0%, 1750/7613 patients) [5] and in ICUs in Southern Europe, Turkey and Iran (23.5%, 176/749 patients) [7] and much higher than rate of 9.1% (156/1707) in ICUs in the United States in 2011[4]. These differences can be explained by several factors: There were few single rooms in ICUs and common rooms usually contain 4–5 beds and even up to 30 beds; In addition with low healthcare staff ratios [11] and high bed occupancy rates [22], often over 110%, makes successful infection control very challenging. The lack of nursing staff for patient care allows little time for proper infection control measures, which may lead to an increased HAI rate [23–25]. This is supported by the smaller amount of alcohol hand rub used: a median of 66.4 ml (IQR: 23.7–100.5 ml)/patient day, compared with a median of 83 ml (IQR: 64–105)/patient day in ICUs of Germany in 2010 [26]. However, the HAI prevalence in Vietnamese ICUs was lower than the pooled prevalence of 35.2% in other LMICs [3].

Hospital-acquired pneumonia (HAP) was the most common type of HAI as in other studies [1,3,7]. HAP accounted for 79.4% of all HAIs in our study, nearly double that reported (40.0% - 45.3%) in ICUs of developed countries [5,6]. This is due to a large proportion of HAP (37.4%, 301/804) was acquired in the same hospital before admission to ICU. This figure raises the need for effective infection control program outside ICU for HAP prevention. Blood stream infections (BSI) accounted for 4.4% of all HAI, considerable lower than the reported 18.0% in European ICUs [5]. Potentially this is due to the high HAP prevalence, but there might also be an underestimation of BSI due to underutilization of blood cultures; as an indication there were a total of only 243 blood culture samples taken from the 3287 patients in the 24 hour preceding the survey day, we are not aware of any comparable data from other settings.

Multivariate analysis identified intubation as an important risk factor, which combined with the high prevalence of HAP suggests that future interventions should target this risk factor. Potential interventions to assess include ventilation and sedation strategies, cuff and tracheal tube design, cuff pressure management [27] Strategies like selective gut decontamination using polymyxins may not be appropriate in this setting with high antimicrobial resistance background rates with colistin as last resort drug [28]. Multivariate analysis revealed that the involvement of the patient’s family in patient care was a protective factor from HAI. As ICUs had department-wide policies to either allow or forbid family from taking care of their admitted family member this practice was highly clustered at the ICU level, the result needs to be interpreted with caution. Further research is needed to understand the risks for HAI regarding family members involved in patient care. National surveillance systems for HAI are scarce in LMIC settings yet ongoing surveillance is crucial to informing policymakers of the needs of the population and high quality data is critical to the development of potential interventions applicable at the national level.

The proportion of HAIs related to medical devices were higher than that reported by a European survey in 2011–2012, where device related HAIs were 59.5% for UTI, 57.3% for primary BSI, and 33.2% for HAP [5]. This may be partly explained by less compliance with hand hygiene [25] and low alcohol hand rub consumption, but probably also sub-optimal routines for insertion and care of urinary and blood catheters.

Nearly 85% of the ICU patients were on antimicrobials in this study, higher than the 56.5% and 77.3% reported in European and American ICUs, respectively [5,29]. Broad-spectrum beta-lactam antibiotics (3^rd^, 4^th^ generation cephalosporins and carbapenems) and fluoroquinolones accounted for 39.7% and 19.4% of total antimicrobials use in Vietnamese ICUs, respectively. These proportions are higher than those reported by European ICUs of around 30% and 10% respectively [5]. Also combination therapy in our study is more common than in European ICUs: 62.5% versus 47.7% [5].

Gram-negative bacteria were the most common cause of HAI, similar to studies in mainly low and middle income countries [3,7,23]. Furthermore, the proportion of infection caused by *Acinetobacter* in ICUs was highest in Asia (19.2%), which was more than five times in compared with North America (3.7%) [30]. The two most frequently isolated bacteria were *A*. *baumannii* and *P*. *aeruginosa* (accounted for 38.2% of total isolates), similar to a study in Turkey in 2007–2008 (34.1% of isolates) [31]. Carbapenem resistance in *A*. *baumannii*, *P*. *aeruginosa*, and *K*. *pneumoniae* in this study was higher than rates of 69.1%, 44.4% and 2.9%, respectively, in Vietnam in 2007 and 2008 [25]. High prevalence of carbapenem resistance leads to an increased colistin use [9] and emergence and spread of colistin resistance will follow. Antimicrobial stewardship programmes and the deployment of alternatives to carbapenems may help to slow the development of further resistance to the remaining active antibiotics [32] and need to be evaluated in this context. The high rates of MRSA and glycopeptide resistant enterococci are also a major concern. However, the burden of Gram-positive infections as a cause of HAI is relatively low as compared to Gram-negative infections.

A potential limitation of this study is the paucity of microbiology data for many HAIs due to the few cultures taken in routine clinical care. However the microbiology results available represent valuable data for assessing the microorganisms causing HAIs and its susceptibility to antimicrobials in our setting. As HAIs had not previously been routinely surveyed in Vietnamese hospitals, criteria for the diagnosis of specific HAI may not have been fully understood, potentially leading to misclassification of specific HAIs. To limit this problem, we organized several workshops and site visits to train doctors. The enrolment of patients who were just admitted to ICU before 8.00 A.M. then were discharged from ICU soon after survey time could lead to underestimate the HAI prevalence. However, the mean ICU stays were much longer (4.8 to 16 days, Table A in S1 File) means that number of these cases was not many and its impact on the HAI prevalence was not significant. HAIs also are a sensitive issue in Vietnam; therefore ICU doctors may not have reported all HAIs, leading to an underestimation. To minimize this effect data, hospitals were anonymized using codes. When data were uploaded we ran data checks and returned queries to doctors that needed to be resolved. We therefore think that the hands-on supervision and input from the study team ensured that data quality was optimal.

## Conclusions

High prevalence of HAIs in Vietnamese ICUs, mainly caused by Gram-negative bacteria with high rates of carbapenem resistance, and high levels of antimicrobial use illustrate the urgent need for capacity strengthening in both rational antimicrobial use and infection control efforts at national, regional and local levels.

## Supporting Information

S1 File Supplementary Tables. Hospital and ICU Characteristics (Table A), Patient Characteristics (Table B), HAI Prevalence per Month (Table C), Antimicrobials Combinations Used (Table D), The Common Antimicrobial Agents Used (Table E)
(DOCX)
