# Supplementary material for: Burden of Hospital Acquired Infections and Antimicrobial Use in Vietnamese Adult Intensive Care Units
Source: PLoS One. 2016 Jan 29;11(1):e0147544. doi: 10.1371/journal.pone.0147544 (PMC4732823; doi:10.1371/journal.pone.0147544)
Supplement: S1 File — Hospital and ICU Characteristics (Table A), Patient Characteristics (Table B), HAI Prevalence per Month (Table C), Antimicrobials Combinations Used (Table D), The Common Antimicrobial Agents Used (Table E) (DOCX) [file pone.0147544.s001.docx]

| Basic hospital structure | | | | | | | Basic participating ICU structure | | | | | | | | | | | | |
| --- | --- | --- | --- | --- | --- | --- | --- | --- | --- | --- | --- | --- | --- | --- | --- | --- | --- | --- | --- |
| Code | Total bed | Parients No. /year | Patient days /year | Alcohol hand rub/year (litres) | Infection control | | Code | Patients No. /year | Patient days /year | Mean ICU stay (day) | Alcohol hand rub/year (litres) | Alcohol/patient day (ml) | Total beds | No. Room | Single room | No. doctors | No. nurses | Doctor/bed | Nurse/Bed |
|  |  |  |  |  | Nurses | Doctors |  |  |  |  |  |  |  |  |  |  |  |  |  |
| H01 | 280 | 7,365 | 76,342 | 1,030 | 0 | 2 | I-ICU | 497 | 4,631 | 9.3 | 130 | 28.1 | 18 | 6 | 2 | 4 | 22 | 0.22 | 1.22 |
| H02 | 1900 | 114,000 | 1,290,000 | 6,152 | 12 | 2 | G-ICU | 1,345 | 14,007 | 10.4 | 1,408 | 100.5 | 38 | 12 | 6 | 13 | 55 | 0.34 | 1.45 |
| H04 | 841 | 42,074 | 287,323 | 400 | 4 | 2 | M-ICU | 1,240 | 5,904 | 4.8 | 140 | 16.9 | 20 | 4 | 0 | 7 | 25 | 0.35 | 1.25 |
|  |  |  |  |  |  |  | S-ICU | 1,228 | 5,904 | 4.8 | 100 | 23.7 | 17 | 4 | 0 | 4 | 24 | 0.24 | 1.41 |
| H05 | 1050 | 41,106 | 366,828 | 1,200 | 29 | 11 | S-ICU | 696 | 5,064 | 7.3 | 336 | 66.4 | 16 | 2 | 0 | 4 | 40 | 0.25 | 2.50 |
| H06 | 900 | 41,430 | 356,356 | 3,500 | 0 | 1 | M-ICU | 1,052 | 5,704 | 5.4 | 500 | 87.7 | 16 | 5 | 0 | 11 | 24 | 0.69 | 1.50 |
| H07 | 750 | 34,848 | 183,885 | 2,996 | 2 | 1 | G-ICU | 751 | 3,508 | 4.7 | 482 | 137.4 | 10 | 4 | 0 | 7 | 18 | 0.70 | 1.80 |
| H08 | 1050 | 15,000 | 60,000 | 1,000 | 12 | 6 | M-ICU | 1,674 | 6,418 | 3.8 | 480 | 74.8 | 20 | 5 | 0 | 5 | 22 | 0.25 | 1.10 |
| H09 | 1500 | 85,054 | 712,844 | 5640 | 25 | 1 | G-ICU | 3,049 | 20,772 | 6.8 | 2,210 | 106.4 | 47 | 7 | 0 | 13 | 38 | 0.28 | 0.81 |
| H10 | 2362 | 85,608 | 891,908 | 10,500 | 10 | 2 | M-ICU | 2,530 | 16,280 | 6.4 | 1,200 | 73.7 | 60 | 7 | 6 | 11 | 66 | 0.18 | 1.10 |
| H11 | 1000 | 57,523 | 373,910 | 650 | 6 | 0 | M-ICU | 2,356 | 12,323 | 5.2 | 480 | 39.0 | 34 | 1 | 0 | 15 | 48 | 0.44 | 1.41 |
| H12 | 750 | 48,049 | 290,215 | 110 | 2 | 1 | G-ICU | 2,374 | 12,218 | 5.1 | 110 | 9.0 | 20 | 4 | 0 | 9 | 32 | 0.45 | 1.60 |
| H13 | 1800 | 123,850 | 918,026 | 13,066 | 3 | 2 | G-ICU | 1,196 | 11,901 | 10.0 | 500 | 42.0 | 31 | 3 | 0 | 15 | 54 | 0.48 | 1.74 |
| H15 | 550 | 41,751 | 288,696 | 1,113 | 3 | 1 | I-ICU | 540 | 8,651 | 16.0 | 900 | 104.0 | 23 | 5 | 1 | 8 | 48 | 0.35 | 2.09 |
| H16 | 800 | 49,248 | 326,723 | 7,200 | 5 | 1 | G-ICU | 2,042 | 18,786 | 9.2 | 360 | 19.2 | 30 | 1 | 0 | 16 | 33 | 0.53 | 1.10 |

**Table A. Hospital and ICU Characteristics**

**Table B. Patient Characteristics**

| Characteristic | | Tertiary hospitals | | | | | | | Province hospitals | | | | | | | | Total |
| --- | --- | --- | --- | --- | --- | --- | --- | --- | --- | --- | --- | --- | --- | --- | --- | --- | --- |
| Hospital | | H01 | H02 | H05 | H07 | H10 | H13 | H16 | H04 | H04 | H06 | H08 | H09 | H11 | H12 | H15 |  |
| ICU type | | I-ICU | G-ICU | S-ICU | G-ICU | G-ICU | G-ICU | G-ICU | S-ICU | M-ICU | M-ICU | M-ICU | G-ICU | M-ICU | G-ICU | I-ICU |  |
| Total patients enrolled, n | | 203 | 223 | 138 | 145 | 578 | 277 | 214 | 89 | 54 | 148 | 187 | 403 | 223 | 223 | 182 | 3287 |
| Age (years) | Mean (median, IQR) | 52.5 (53, 43 – 65) | 54.7 (57, 39 – 71) | 47.5 (47, 27 – 64.2) | 59.2 (64, 45.5 – 76) | 56.5 (56, 40 – 76) | 59.6 (64, 44 – 76) | 68.3 (72, 57.7 – 83) | 54.1 (57, 38 – 70) | 70.7 (73, 62 – 81) | 65.8 (68, 54.5 – 79.7) | 67.3 (73, 53 – 79) | 64.5 (68, 50 – 80) | 60.8 (63, 48 – 78) | 57.8 (59, 37 – 80) | 51.9 (54, 35 – 65) | 59.2 (61, 45 – 77) |
|  | Missing | 0 | 0 | 0 | 0 | 0 | 0 | 0 | 0 | 0 | 0 | 0 | 0 | 0 | 0 | 0 | 0 |
| Gender, n (%) | Male | 145 (71.4) | 145 (65) | 111 (80.4) | 114 (78.6) | 415 (71.8) | 145 (52.3) | 111 (51.9) | 61 (68.5) | 31 (57.4) | 96 (64.9) | 118 (63.1) | 210 (52.1) | 138 (61.9) | 143 (64.1) | 118 (64.8) | 2101 (63.9) |
|  | Female | 58 (28.6) | 78 (35) | 27 (19.6) | 31 (21.4) | 163 (28.2) | 132 (47.7) | 103 (48.1) | 28 (31.5) | 23 (42.6) | 52 (35.1) | 69 (36.9) | 193 (47.9) | 85 (38.1) | 80 (35.9) | 64 (35.2) | 1186 (36.1) |
|  | Missing | 0 | 0 | 0 | 0 | 0 | 0 | 0 | 0 | 0 | 0 | 0 | 0 | 0 | 0 | 0 | 0 |
| Comorbidity, n (%) | Yes | 82 (40.6) | 131 (59.5) | 8 (5.8) | 87 (60) | 107 (18.5) | 121 (43.7) | 126 (59.1) | 28 (31.5) | 34 (63) | 104 (70.3) | 93 (49.7) | 141 (37) | 43 (36.4) | 83 (37.2) | 61(30.1) | 1249 (39.6) |
|  | No | 120 (59.4) | 89 (40.5) | 129 (94.2) | 58 (40) | 471 (81.5) | 156 (56.3) | 87 (40.9) | 61 (68.5) | 20 (37) | 44 (29.7) | 94 (50.3) | 240 (63) | 75 (63.6) | 140 (62.8) | 118 (69.9) | 1902 (60.4) |
|  | Missing | 1 | 3 | 1 | 0 | 0 | 0 | 1 | 0 | 0 | 0 | 0 | 22 | 105 | 0 | 3 | 136 |
| Source of ICU admission, n (%) | Community | 0 | 59 (26.8) | 10 (10.1) | 78 (53.8) | 424 (73.4) | 19 (6.9) | 79 (36.9) | 26 (29.2) | 47 (87) | 99 (67.3) | 102 (54.5) | 207 (53.6) | 23 (28.7) | 197 (89.1) | 57 (31.8) | 1427 (46.2) |
|  | Current hospital | 115 (57.2) | 85 (38.6) | 94 (85.5) | 40 (27.6) | 90 (15.6) | 159 (57.4) | 66 (30.8) | 27 (30.3) | 3 (5.6) | 37 (25.2) | 45 (24.1) | 101 (26.2) | 32 (40) | 16 (7.2) | 20 (11.2) | 930 (30.1) |
|  | Other hospital | 86 (42.8) | 76 (34.5) | 6 (5.5) | 27 (18.6) | 64 (11.1) | 96 (34.7) | 69 (32.2) | 22 (24.7) | 3 (5.6) | 8 (5.4) | 40 (21.4) | 26 (6.7) | 8 (10) | 6 (2.7) | 101 (56.4) | 638 (20.7) |
|  | Other | 0 | 0 | 0 | 0 | 0 | 3 (1.1) | 0 | 14 (15.7) | 1 (1.9) | 3 (2.0) | 0 | 52 (13.5) | 17 (21.3) | 2 (0.9) | 1 (0.6) | 93 (3) |
|  | Missing | 2 | 3 | 28 | 0 | 0 | 0 | 0 | 0 | 0 | 1 | 0 | 17 | 143 | 2 | 3 | 199 |
| Reason for admission, n (%) | Medical | 1 (0.5) | 82 (37.3) | 3 (2.8) | 57 (39.3) | 368 (63.7) | 32 (11.6) | 100 (46.7) | 11 (12.6) | 31 (57.4) | 99 (68.3) | 46 (24.7) | 189 (49.7) | 42 (52.5) | 139 (63.2) | 3 (1.7) | 1203 (39.1) |
|  | Infection | 200 (99.5) | 123 (55.9) | 6 (5.5) | 37 (25.5) | 88 (15.2) | 159 (57.4) | 114 (53.3) | 13 (14.9) | 23 (42.6) | 46 (31.7) | 140 (75.3) | 188 (49.5) | 31 (38.8) | 81 (36.8) | 174 (97.8) | 1423 (46.3) |
|  | Surgery | 0 | 15 (6.8) | 100 (91.7) | 51 (35.2) | 122 (21.1) | 86 (31) | 0 | 63 (72.4) | 0 | 0 | 0 | 3 (0.8) | 7 (8.8) | 0 | 1 (0.6) | 448 (14.6) |
|  | Missing | 2 | 3 | 29 | 0 | 0 | 0 | 0 | 2 | 0 | 3 | 1 | 23 | 143 | 3 | 4 | 213 |
| Surgery since admission, n (%) | None | 118 (58.1) | 160 (72.7) | 4 (2.9) | 94 (64.8) | 541 (93.6) | 127 (45.8) | 156 (72.9) | 28 (31.5) | 52 (96.3) | 136 (91.9) | 186 (99.5) | 375 (93.1) | 154 (69.1) | 218 (97.8) | 121 (66.5) | 2470 (75.2) |
|  | Minor | 85 (41.9) | 31 (14.1) | 0 | 5 (3.4) | 3 (0.5) | 43 (15.5) | 29 (13.6) | 3 (3.4) | 1 (1.9) | 9 (6.1) | 1 (0.5) | 9 (2.2) | 45 (20.2) | 2 (0.9) | 61 (33.5) | 327 (10) |
|  | Major | 0 | 29 (13.2) | 134 (97.1) | 46 (31.7) | 34 (5.9) | 107 (38.6) | 29 (13.6) | 58 (65.2) | 1 (1.9) | 3 (2.0) | 0 | 19 (4.7) | 24 (10.8) | 3 (1.3) | 0 | 487 (14.8) |
|  | Missing | 0 | 3 | 0 | 0 | 0 | 0 | 0 | 0 | 0 | 0 | 0 | 0 | 0 | 0 | 0 | 3 |
| Intubation, n (%) | Yes | 165 (81.3) | 98 (43.9) | 138 (100) | 81 (55.9) | 184 (31.8) | 241 (87) | 160 (74.8) | 45 (50.6) | 7 (13) | 76 (51.4) | 117 (62.6) | 189 (46.9) | 92 (41.3) | 50 (22.4) | 76 (41.8) | 1719 (52.3) |
|  | No | 38 (18.7) | 125 (56.1) | 0 | 64 (44.1) | 394 (68.2) | 36 (13) | 54 (25.2) | 44 (49.4) | 47 (87) | 72 (48.6) | 70 (37.4) | 214 (53.1) | 131 (58.7) | 173 (77.6) | 106 (58.2) | 1568 (47.7) |
|  | Missing | 0 | 0 | 0 | 0 | 0 | 0 | 0 | 0 | 0 | 0 | 0 | 0 | 0 | 0 | 0 | 0 |
| Central vascular catheter, n (%) | Yes | 99 (48.8) | 129 (57.9) | 133 (96.4) | 13 (9) | 49 (8.5) | 213 (76.9) | 31 (14.5) | 44 (49.4) | 3 (5.6) | 38 (25.7) | 7 (3.7) | 79 (19.6) | 17 (7.6) | 31 (13.9) | 35 (19.2) | 921 (28) |
|  | No | 104 (51.2) | 94 (42.1) | 5 (3.6) | 132 (91) | 529 (91.5) | 64 (23.1) | 183 (85.5) | 45 (50.6) | 51 (94.4) | 110 (74.3) | 180 (96.3) | 324 (80.4) | 206 (92.4) | 192 (86.1) | 147 (80.8) | 2366 (72) |
|  | Missing | 0 | 0 | 0 | 0 | 0 | 0 | 0 | 0 | 0 | 0 | 0 | 0 | 0 | 0 | 0 | 0 |
| Urinary catheter, n (%) | Yes | 170 (83.7) | 133 (59.6) | 137 (99.3) | 65 (44.8) | 71 (12.3) | 246 (88.8) | 107 (50) | 69 (77.5) | 11 (20.4) | 51 (34.5) | 33 (17.6) | 265 (65.8) | 68 (30.5) | 128 (57.4) | 62 (34.1) | 1616 (49.2) |
|  | No | 33 (16.3) | 90 (40.4) | 1 (0.7) | 80 (55.2) | 507 (87.7) | 31 (11.2) | 107 (50) | 20 (22.5) | 43 (79.6) | 97 (65.5) | 154 (82.4) | 138 (34.2) | 155 (69.5) | 95 (42.6) | 120 (65.9) | 1671 (50.8) |
|  | Missing | 0 | 0 | 0 | 0 | 0 | 0 | 0 | 0 | 0 | 0 | 0 | 0 | 0 | 0 | 0 | 0 |
| Peripheral vascular catheter, n (%) | Yes | 126 (62.1) | 188 (84.3) | 5 (3.6) | 106 (73.1) | 532 (92) | 182 (65.7) | 213 (99.5) | 67 (75.3) | 45 (83.3) | 100 (67.6) | 169 (90.4) | 391 (97) | 216 (96.9) | 217 (97.3) | 166 (91.2) | 2723 (82.8) |
|  | No | 77 (37.9) | 35 (15.7) | 133 (96.4) | 39 (26.9) | 46 (8) | 95 (34.3) | 1 (0.5) | 22 (24.7) | 9 (17.6) | 48 (32.4) | 18 (9.6) | 12 (3) | 7 (3.1) | 6 (2.7) | 16 (8.8) | 564 (17.2) |
|  | Missing | 0 | 0 | 0 | 0 | 0 | 0 | 0 | 0 | 0 | 0 | 0 | 0 | 0 | 0 | 0 | 0 |
| Dialysis, n (%) | Yes | 13 (6.4) | 75 (33.6) | 0 | 9 (6.2) | 4 (0.7) | 57 (20.6) | 25 (11.7) | 0 | 0 | 19 (12.8) | 2 (1.1) | 21 (5.2) | 34 (15.2) | 7 (3.1) | 4 (2.2) | 270 (82) |
|  | No | 190 (93.6) | 148 (66.4) | 138 (100) | 136 (93.8) | 574 (99.3) | 220 (79.4) | 189 (88.3) | 89 (100) | 54 (100) | 129 (87.2) | 185 (98.9) | 382 (94.8) | 189 (84.8) | 216 (96.9) | 178 (97.8) | 3017 (91.8) |
|  | Missing | 0 | 0 | 0 | 0 | 0 | 0 | 0 | 0 | 0 | 0 | 0 | 0 | 0 | 0 | 0 | 0 |
| Family member supporting a part in patient care, n (%) | Yes | 198 (97.5 | 220 (98.7) | 2 (1.4) | 142 (97.9) | 573 (99.1) | 6 (2.2) | 2 (0.9) | 89 (100) | 54 (100) | 148 (100) | 187 (100) | 5 (1.2) | 222 (99.6) | 221 (99.1) | 3 (1.6) | 2072 (63.0) |
|  | No | 5 (2.5) | 3 (1.3) | 136 (98.6) | 3 (2.1) | 5 (0.9) | 271 (97.8) | 212 (99.1) | 0 | 0 | 0 | 0 | 398 (98.8) | 1 (0.4) | 2 (0.9) | 179 (98.4) | 1215 (37.0) |
| Antibiotic use, n (%) | Yes | 155 (76.4) | 184 (82.5) | 134 (97.1) | 89 (61.4) | 577 (99.8) | 250 (90.3) | 175 (81.8) | 83 (93.3) | 27 (50) | 131 (88.5) | 148 (79.1) | 383 (95) | 188 (84.3) | 136 (61) | 127 (69.8) | 2787 (84.8) |
|  | No | 48 (23.6) | 39 (17.5) | 4 (2.9) | 56 (38.6) | 1 (0.2) | 27 (9.7) | 39 (18.2) | 6 (6.7) | 27 (50) | 17 (11.5) | 39 (20.9) | 20(5) | 35 (15.7) | 87 (39) | 55 (30.2) | 500 (15.2) |
|  | Missing | 0 | 0 | 0 | 0 | 0 | 0 | 0 | 0 | 0 | 0 | 0 | 0 | 0 | 0 | 0 | 0 |
| Hospital acquired infection, n (%) | Yes | 68 (33.5) | 84 (38.5) | 84 (60.9) | 21 (14.5) | 40 (6.9) | 140 (50.5) | 126 (59.2) | 12 (13.5) | 3 (5.6) | 43 (30.5) | 40 (21.4) | 146 (36.8) | 81 (36.5) | 36 (16.1) | 41 (22.7) | 965 (29.5) |
|  | No | 135 (66.5) | 134 (61.5) | 54 (39.1) | 124 (85.5) | 538 (93.1) | 137 (49.5) | 87 (40.8) | 77 (86.5) | 51 (94.4) | 98 (69.5) | 147 (78.6) | 251 (63.2) | 141 (63.5) | 187 (83.9) | 140 (77.3) | 2301 (70.5) |
|  | Missing | 0 | 5 | 0 | 0 | 0 | 0 | 1 | 0 | 0 | 7 | 0 | 6 | 1 | 0 | 1 | 21 |

**Table C. HAI Prevalence per Month**

| **Patients** | **Month survey** | | | | | | | | | | | | **Total** |
| --- | --- | --- | --- | --- | --- | --- | --- | --- | --- | --- | --- | --- | --- |
|  | January | February | March | April | May | June | July | August | September | October | November | December |  |
| **HAI patients, n (%)** | 76 (28.1) | 84 (28.4) | 124 (34.1) | 72 (23.8) | 105 (30.3) | 83 (26.2) | 111 (33.4) | 82 (25.5) | 85 (28.2) | 15 (31.9) | 32 (34.8) | 96 (34.7) | 965 (29.5) |
| **Total patients, n** | 270 | 296 | 364 | 302 | 347 | 317 | 332 | 321 | 301 | 47 | 92 | 277 | 3266 |

**Table D. Antimicrobials Combinations**

| **Antimicrobials combinations in patients used 2 antimicrobials** | | | **Antimicrobials combinations in patients used 3 antimicrobials** | | | | **Antimicrobials combinations in patients used 4 antimicrobials** | | | | |
| --- | --- | --- | --- | --- | --- | --- | --- | --- | --- | --- | --- |
| Antimicrobial combinations  (n = 1343) | | % (n) | Antimicrobial combinations (n = 552) | | | % (n) | Antimicrobial combinations (n=159) | | | | % (n) |
| J01DD Third-generation cephalosporins | J01MA Fluoroquinolones | 19.1 (257) | J01DD Third-generation cephalosporins | J01MA Fluoroquinolones | J01XA Glycopeptide antibacterials | 13.6 (75) | J01DE Fourth-generation cephalosporins | J01GB Other aminoglycosides | J01MA Fluoroquinolones | J01XA Glycopeptide antibacterials | 13.2 (21) |
| J01DH Carbapenems | J01MA Fluoroquinolones | 10.8 (145) | J01DD Third-generation cephalosporins | J01MA Fluoroquinolones | J01XD Imidazole derivatives | 6.0 (33) | J01DD Third-generation cephalosporins | J01GB Other aminoglycosides | J01MA Fluoroquinolones | J01XA Glycopeptide antibacterials | 10.7 (17) |
| J01DD Third-generation cephalosporins | J01GB Other aminoglycosides | 7.8 (105) | J01DH Carbapenems | J01MA Fluoroquinolones | J01XA Glycopeptide antibacterials | 6.0 (33) | J01CR Combinations of penicillins included beta-lactamase inhibitors | J01DD Third-generation cephalosporins | J01MA Fluoroquinolones | J01XA Glycopeptide antibacterials | 3.1 (5) |
| J01DH Carbapenems | J01XA Glycopeptide antibacterials | 5.0 (67) | J01DD Third-generation cephalosporins | J01GB Other aminoglycosides | J01MA Fluoroquinolones | 5.6 (31) | J01DC Second-generation cephalosporins | J01GB Other aminoglycosides | J01MA Fluoroquinolones | J01XA Glycopeptide antibacterials | 2.5 (4) |
| J01DE Fourth-generation cephalosporins | J01MA Fluoroquinolones | 4.8 (65) | J01DE Fourth-generation cephalosporins | J01MA Fluoroquinolones | J01XA Glycopeptide antibacterials | 4.9 (27) | J01DD Third-generation cephalosporins | J01FA Macrolides | J01MA Fluoroquinolones | J01XA Glycopeptide antibacterials | 2.5 (4) |
| J01DH Carbapenems | J01XB Polymyxins (colistin injection) | 4.5 (61) | J01DD Third-generation cephalosporins | J01GB Other aminoglycosides | J01XA Glycopeptide antibacterials | 2.9 (16) | J01DD Third-generation cephalosporins | J01MA Fluoroquinolones | J01XA Glycopeptide antibacterials | J01XD Imidazole derivatives | 2.5 (4) |
| J01DH Carbapenems | J01XD Imidazole derivatives | 4.4 (59) | J01DE Fourth-generation cephalosporins | J01GB Other aminoglycosides | J01MA Fluoroquinolones | 2.5 (14) | J01DH Carbapenems | J01GB Other aminoglycosides | J01MA Fluoroquinolones | J01XA Glycopeptide antibacterials | 2.5 (4) |
| J01DD Third-generation cephalosporins | J01XD Imidazole derivatives | 4.2 (57) | J01DH Carbapenems | J01MA Fluoroquinolones | J01XD Imidazole derivatives | 2.5 (14) | J01CR Combinations of penicillins included beta-lactamase inhibitors | J01DE Fourth-generation cephalosporins | J01FA Macrolides | J01MA Fluoroquinolones | 1.9 (3) |
| J01DH Carbapenems | J01XX Other antibacterials | 3.4 (46) | J01DH Carbapenems | J01XA Glycopeptide antibacterials | J01XD Imidazole derivatives | 2.2 (12) | J01CR Combinations of penicillins included beta-lactamase inhibitors | J01DE Fourth-generation cephalosporins | J01MA Fluoroquinolones | J01XA Glycopeptide antibacterials | 1.9 (3) |
| J01DH Carbapenems | J01GB Other aminoglycosides | 2.8 (38) | J01DH Carbapenems | J01XA Glycopeptide antibacterials | J01XB Polymyxins | 2.0 (11) | J01DE Fourth-generation cephalosporins | J01FA Macrolides | J01GB Other aminoglycosides | J01XA Glycopeptide antibacterials | 1.9 (3) |
| Other 91 combinations | | 33.0 (443) | Other 137 combinations | | | 51.8 (286) | Other 77 combinations | | | | 57.2 (97) |

**Table E. The Common Antimicrobial Agents Used**

| Patients used two antimicrobials  (n = 1343 patient) | | Patients used three antimicrobials  (n = 552 patients) | | Patients used four antimicrobials  (n = 159 patients) | | Total antimicrobials used (n=5590) | |
| --- | --- | --- | --- | --- | --- | --- | --- |
| Antimicrobial group | % (n) | Antimicrobial group | % (n) | Antimicrobial group | % (n) | Antimicrobial group | % (n) |
| J01MA Fluoroquinolones | 21.1 (556) | J01MA Fluoroquinolones | 21.1 (350) | J01MA Fluoroquinolones | 19.3 (123) | J01DD Third-generation cephalosporins | 20.1 (1126) |
| J01DD Third-generation cephalosporins | 19.8 (533) | J01XA Glycopeptide antibacterials | 15.5 (257) | J01XA Glycopeptide antibacterials | 17.3 (110) | J01MA Fluoroquinolones | 19.4 (1082) |
| J01DH Carbapenems | 16.6 (445) | J01DD Third-generation cephalosporins | 13.6 (225) | J01GB Other aminoglycosides | 15.1 (96) | J01DH Carbapenems | 14.1 (786) |
| J01GB Other aminoglycosides | 9.2 (247) | J01GB Other aminoglycosides | 10.3 (171) | J01DD Third-generation cephalosporins | 8.8 (56) | J01XA Glycopeptide antibacterials | 9.6 (536) |
| J01XA Glycopeptide antibacterials | 5.7 (153) | J01DH Carbapenems | 10.3 (170) | J01DE Fourth-generation cephalosporins | 8.6 (55) | J01GB Other aminoglycosides | 9.3 (521) |
| J01XD Imidazole derivatives | 5.5 (149) | J01XD Imidazole derivatives | 6.4 (106) | J01DH Carbapenems | 5.8 (37) | J01DE Fourth-generation cephalosporins | 5.5 (310) |
| J01DE Fourth-generation cephalosporins | 4.4 (118) | J01DE Fourth-generation cephalosporins | 5.3 (87) | J01XD Imidazole derivatives | 4.6 (29) | J01XD Imidazole derivatives | 5.2 (289) |
| J01XB Polymyxins | 4.2 (112) | J01XB Polymyxins | 3.6 (60) | J01FA Macrolides | 3.9 (25) | J01CR Combinations of penicillins included beta-lactamase inhibitors | 3.5 (194) |
| J01CR Combinations of penicillins included beta-lactamase inhibitors | 3.3 (88) | J01CR Combinations of penicillins included beta-lactamase inhibitors | 1.8 (29) | J01CR Combinations of penicillins included beta-lactamase inhibitors | 3.3 (21) | J01XB Polymyxins | 3.3 (186) |
| J01XX Other antibacterials | 3.1 (83) | J01DC Second-generation cephalosporins | 1.7 (28) | J01DC Second-generation cephalosporins | 2.8 (18) | J01XX Other antibacterials | 2.2 (122) |
| 18 other antibacterial groups | 7.1 (192) | 18 other antibacterial groups | 10.4 (173) | 16 other 16 antibacterial groups | 10.4 (66) | 12 other antibacterial groups | 7.8 (438) |
